# Assessment of a New Medical Device (Pirifix^TM^) for Positioning and Maintaining the Upper Dental Arch during Le Fort I Osteotomy

**DOI:** 10.3390/jpm14030324

**Published:** 2024-03-20

**Authors:** Pierre-Etienne Serree, Eugénie Bertin, Camille Coussens, Eleonore Brumpt, Jean-François Devoti, Aurélien Louvrier

**Affiliations:** 1 Chirurgie Maxillo-Faciale, Stomatologie et Odontologie Hospitalière, CHU Besançon, Université de Franche-Comté, F-25000 Besançon, France; ebertin@chu-besancon.fr (E.B.); ebrumpt@chu-besancon.fr (E.B.); alouvrier@chu-besancon.fr (A.L.); 2 SINERGIES, Université de Franche-Comté, F-25000 Besançon, France; ccoussens@chu-besancon.fr; 3 Plateforme I3DM (Impression 3D Médicale), CHU Besançon, Université de Franche-Comté, F-25000 Besançon, France; 4 CHU Besançon, Radiologie, Université de Franche-Comté, F-25000 Besançon, France; 5 Service de Chirurgie Maxillo-Faciale, Plastique, Reconstructrice et Esthétique, CHU Nancy, Université de Lorraine, F-54000 Nancy, France; j.devoti@chru-nancy.fr

**Keywords:** computer-aided design, equipment and supplies, orthognathic surgery, 3D printing

## Abstract

Introduction: Several medical devices (MDs) are used to assist surgeons in positioning the upper dental arch (UDA) during Le Fort I osteotomies (LFIOs). Some only allow holding, others only positioning. This study aimed to assess the accuracy of a new MD (Pirifix^TM^) coupling these two functions during LFIO on 3D-printed models. Materials and Methods: DICOM data were selected from patients who underwent surgical planning for LFIO between 27 July 2020 and 1 December 2022. Their anatomy was reproduced after segmentation, planning, and stereolithography in two models. Each model was assigned to one of two surgical groups: the control group (positioning by occlusal splint) and the Pirifix^TM^ group. Each patient’s model was planned with the objective of horizontalizing and recentering the UDA. After positioning, models were digitalized using Einscan Pro 2X and compared to the planned model with CloudCompare. The statistical analysis was performed using the Wilcoxon Mann–Whitney test. The result was considered significant if the *p*-value was less than 0.05. Results: Twenty-one patients were selected. Forty-two anatomical models were 3D-printed. The mean difference compared to the planned and corrected positions was 0.69 mm for the control group and 0.84 mm for the Pirifix^TM^ group (*p* = 0.036). Conclusion: Pirifix^TM^ may be a new alternative to available MDs. Further investigations are needed to describe the relationship between the device and facial soft tissues.

## 1. Introduction

Dentoskeletal disharmony is a frequent clinical condition whose etiology may be congenital or acquired. The description of the patient’s facial morphology is based on clinical, 2D, and 3D radiological interpretation [1,2]. Orthognathic surgery complements orthodontics in the management of these disharmonies. It allows dental occlusion disorders to be corrected and aesthetic amelioration and orofacial functions to be improved. When the disharmony involves the maxillary bones, a Le Fort I osteotomy (LFIO) may be warranted. The correction of malposition is based on three axes of translation and three axes of rotation. Most often, it combines several movements, which makes positioning the maxillae more difficult [3,4]. 

Several medical devices (MDs) have been used for positioning the upper dental arch (UDA). These include occlusal splints, spacers [5,6], cutting guides combined with custom-made plates [7,8,9,10,11,12,13,14,15], and intraoperative navigation [10,16,17,18,19,20]. Each technique has limitations. Occlusal splints use a mobile bone, the mandible, as a reference for positioning the upper dental arch. Spacers only help surgeons with vertical dimensions without holding function. Custom-made plates are expensive and require a preoperative CT scan, which is associated with patient irradiation. Splints, guides, and custom-made plates are single-use medical devices with an environmental drawback. Intraoral navigation also requires preoperative planning and implies a specific installation in the operating theatre [16,21]. Furthermore, none of these devices allow for intraoperative adaptation in the same way as the mandibular on-site adjustment plates. All these devices are designed preoperatively and are not adjustable afterward. The assistance they provide becomes limited in complex cases of dentoskeletal disharmony requiring the surgeon to adapt the position of the upper dental arch intraoperatively. A medical device that combines support for the UDA with the ability for the operator to adjust its position would be ideal. The Ennoia company filed a patent in March 2023 for a new MD named Pirifix^TM^, which meets these indications. It meets sterilizable class I MD defined by European regulations [22]. 

This study aimed to assess the precision of Pirifix^TM^ for maintaining and adjusting the position of the upper dental arch during LFIO on models with dentoskeletal disharmony involving the middle third of the face.

## 2. Materials and Methods

### 2.1. Description of the Medical Device

**Figure 1:**
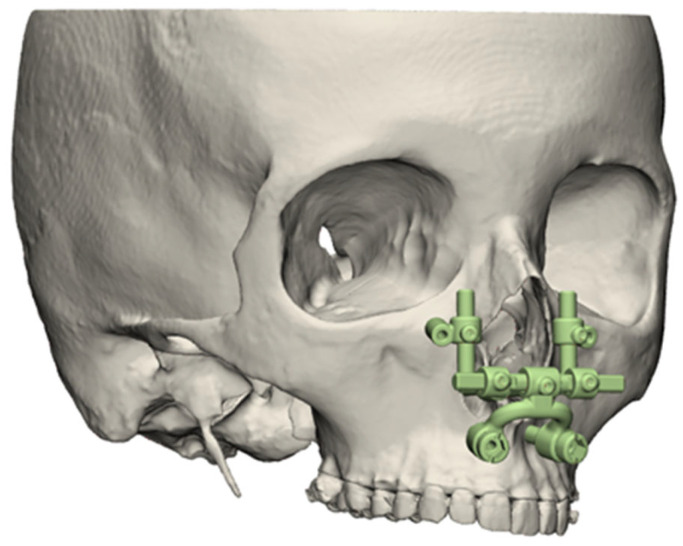
Three-dimensional illustration of Pirifix^TM^ positioning around the piriform orifice.

**Figure 2:**
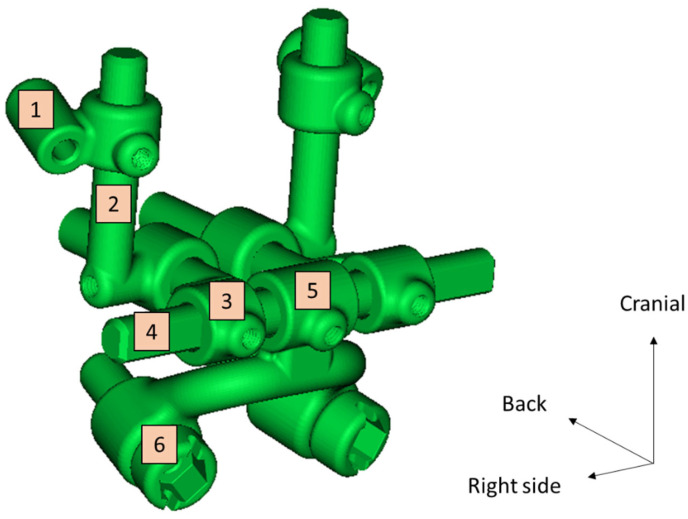
Experimental prototype file of Pirifix^TM^. 1: Right paranasal bone support; 2: right vertical axis; 3: right anteroposterior axis; 4: transverse axis; 5: inverted U-shaped part; 6: right maxillary bone support.

The Pirifix^TM^ device (Ennoïa, Besançon, France) is a bone-supported medical device. Its design is intended to adapt to the anatomy of the perimeter of the lower part of the piriform orifice (Figure 1). It is made up of ten parts produced in biocompatible 17-4ph stainless steel by selective laser melting technology. From top to bottom, the device consists of two paranasal bone-supported parts (right and left), which are associated with two vertical axes (right and left) and articulated with two anteroposterior axes (right and left) joined by a transverse axis (odd and median). This axis is also articulated at its center with an inverted U-shaped arch, with an inferior and posterior concavity. This arch carries the two premaxillary bone-supported parts (Figure 2). The device can be fixed on the skull with four screws (two below the line of an LFIO and two above). The various parts of the device are articulated together so that the palatal bone fragment can be moved in all three planes of space and then held in place. The two vertical axes are used for upward/downward and roll movements. The two anteroposterior axes are used for moving forward. An inverted U-shaped and the transversal axis allow for right or left translation and pitch. All the parts of the device are locked in the desired position by seven screws. 

### 2.2. Creation of an Experimental Model

#### 2.2.1. Data Selection

DICOM data from patients who had virtual planned LFIOs between 27 July 2020 and 1 December 2022 were selected. This corresponds to all patient data archived at the Besançon University Hospital’s 3D-printing platform since its inception. Exclusion criteria were the patient’s opposition to the use of personal data, CT scans without all facial bones, CT scans with less than 300 slices, and no digital dental impression available. Ethical considerations, notably the absence of refusal by patients to use their data, were validated by the Clinical Research and Innovation Department of Besançon University Hospital. Sex, age at CT scan, Angle’s classification and surgical treatments for each patient were noted to describe the population. 

#### 2.2.2. Conception

The models were designed and used on the Besançon University Hospital’s 3D-printing platform. The procedure was repeated for each case included. Skulls and mandibles were segmented from DICOM data using Mimics Medical 25.0 software (Materialise, Leuven, Belgium). Corresponding dental models were added to the file. After a semi-automatic alignment step, segmented teeth were replaced by dental models. Alignment was considered complete when the average distance between the teeth from models and the segmented teeth on the CT scan was less than 0.01 mm.

Parts obtained in standard triangle language (STL) were then modified using 3-Matic 16.0 software (Materialise, Leuven, Belgium). Skull models were positioned on an orthogonal reference frame consisting of the median sagittal plane and the Frankfurt plane. The mandible was translated so far as to close the temporomandibular gap. Then, it was rotated clockwise around the bicondylar axe to remove overlapping between the mandibular and maxillary teeth. The calvaria was removed above a horizontal line passing through the middle of the forehead. The posterior part of the skull was removed behind a frontal plane passing between the foramen magnum and the mastoid processes. This plane was also used to develop a plate for fixing the skull to the table using a rail. An offset of 0.1 mm was applied to this rail, which was then subtracted from the skull by Boolean operation. The optic canals, orbital fissures, infraorbital foramina, and maxillary sinus were preserved. All other holes were filled. Skull surfaces were moderately smoothed.

**Figure 3:**
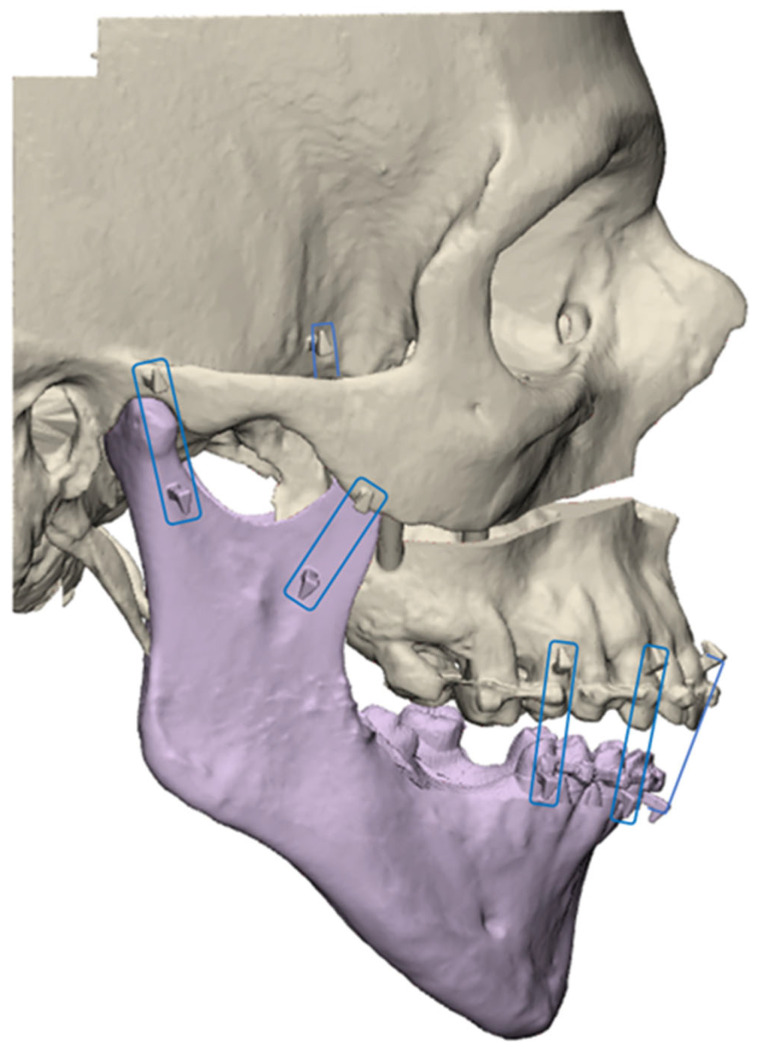
Example of an experimental model associating the mandible (in violet) with the rest of the skull. The pairs of hooks used to articulate the two objects are framed in blue.

Sixteen pairs of hooks were placed on each model. Six pairs reproduced orthodontic brackets. They were placed on the necks of the medial incisors, canines, and first premolars. Two pairs reproduced the resistance of the palatal mucosa. They were placed on the medial pterygoid processes and the posterior palate. Two pairs reproduced the temporomandibular joint capsule. They were placed on the base of the zygomatic arch and the condylar neck. Six pairs reproduced the muscular traction of the masseter, temporal, and medial pterygoid muscles. They were placed at their respective insertions (Figure 3).

**Figure 4:**
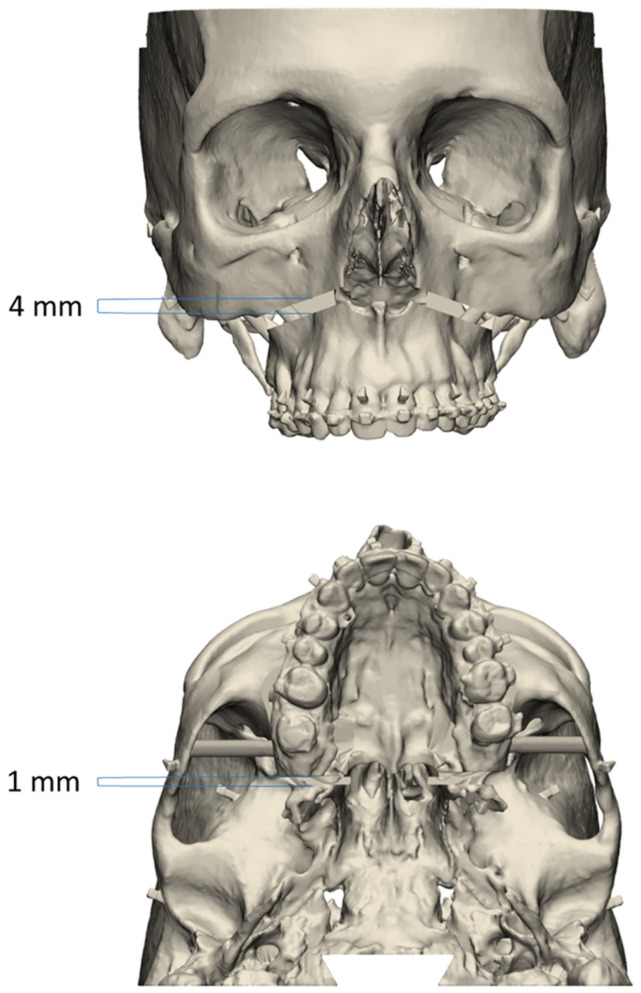
Example of an experimental model with Le Fort I osteotomy and pterygopalatomaxillary disjunction.

LFIO and pterygopalatomaxillary disjunction (PPMD) were simulated using standardized boxes subtracted from the anatomical model. The LFIO box was herringbone-shaped and approximately 4 mm thick. It was centered on the midline. It cut through the vomer bone and maxillary sinus walls. Its posterior part was in continuity with the PPMD box. The PPMD box was a vertical 1 mm thick plane separating the pterygoid processes from the maxillary sinuses (Figure 4). 

**Figure 5:**
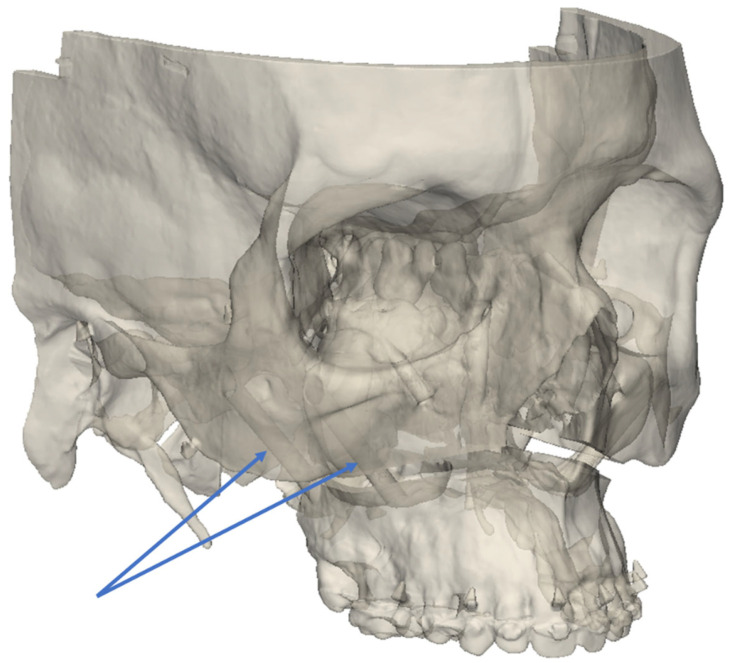
Example of experimental model seen in semi-transparency, showing the right bridges connecting the UDA to the rest of the skull (blue arrows).

The models were completed by adding 4 cylindrical bridges between the UDA and the remaining skull. The right bridges were parallel to each other. The left bridges were also parallel. At the top, the bridges were supported on the internal cortical of the maxillary sinus lateral wall. At the bottom, they were supported on the maxillary sinus floor. The skulls with bridges and mandibles were recorded as two distinct objects for printing (Figure 5). 

#### 2.2.3. Planned Models

No modification of the UDA’s position was applied to files used for printing. These files were also used as a reference to compare the return to the initial position (P0 position). To compare the groups in their ability to place the UDA in the corrected position (C position), a planned (reference) model was made for each patient. With these models, the UDA was horizontalized parallel to the Frankfurt plane. The interincisal point was centered on the median sagittal plane. For each planned model, the distance between the hooks of teeth 13 and 23 and the lowest point of the right and left infraorbital rims was recorded.

#### 2.2.4. Splint Design

For each planned model, an occlusal positioning splint was designed using 3-Matic software. The upper and lower dental arches were circumscribed by a curve using a computer tool. The two curves were converted into a surface, and the space between these two surfaces was filled by constructing a three-dimensional object. An offset of 0.20 mm was applied to the dental arch models before being subtracted from the object by Boolean operation. The result was an arch-shaped splint bearing the maxillary and mandibular dental impressions in the planned position. It was used for P0 positioning (P0 splint) and C positioning (C splint).

#### 2.2.5. Three-Dimensional Printing

**Figure 6:**
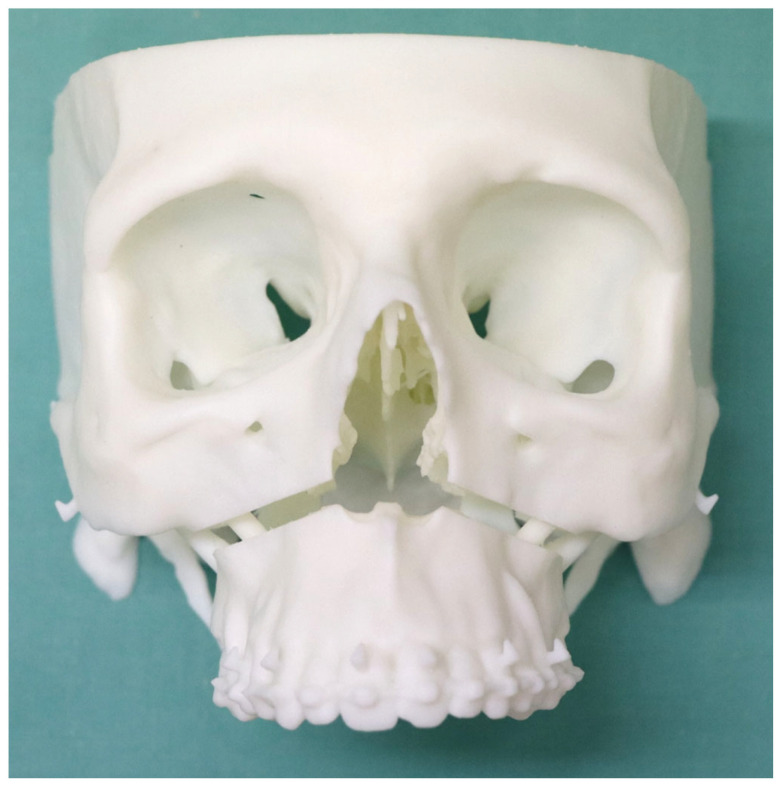
Example of an experimental 3D-printed model.

Stereolithography was used with Preform software (Formlabs, Somerville, MA, USA) and a Form2 or Form3+ printer (Formlabs). The models were printed on a 1:1 scale with white resin (Figure 6), while the splints were printed with white, color base, or clear resin. The supports were manually removed. After, the models needed to be cleaned with a 20 min long isopropanol bath in FormWash (Formlabs) and they were then polymerized for 1 h with FormCure (Formlabs). Two copies of each model were printed. Each copy was assigned to one of two surgical groups: the control group (positioning by occlusal splint) and the Pirifix^TM^ group. The splints were printed using the same protocol as the experimental models and were assigned to the control group.

FDM technology was used to print fixation devices. The slicer software was Cura 4.6 and the printer was an Ultimaker 5 (Ultimaker, Utrecht, Netherlands).

#### 2.2.6. Digitalizing

The models were scanned using an Einscan Pro 2x optical scanner (Shining 3D, Hangzhou, China). The angle between the rotative table and lens was 30°. We captured 30 shots per rotation. Four rotations were completed by changing the position of the models. During each 30 successive picture shot sequences, models were placed on the top edge, the back edge, the right temple, and the left temple. The watertight tool was turned off before making the mesh models. They were then cleaned up with 3-Matic by suppressing free voxels. Digitalizing was performed before UDA separation. After printing and before UDA separation, the printed models were digitalized for the first time and compared to the designed models to describe the printing and digitalizing precision and initial comparability of the groups. 

#### 2.2.7. Comparison

CloudCompare software (v2.13) was used to compare the models. First, they were manually superimposed. Next, we performed automatic matching between these two models based on the frontal, temporal, nasal, and zygomatic bones as the best overlapping areas (UDA segmented). This process was completed according to the root mean square setting at 1 × 10^−7^. After matching, motion vectors were applied to the entire object comprising the UDA. Next, the digitalized models were segmented to only retain dental crowns. The surface was transformed into a cloud of one million points. We performed a cloud-to-mesh (C2M) comparison between the segmented cloud and the planned models as the reference. The absolute difference in millimeters and the standard deviation were noted. 

### 2.3. Evaluation of Device-Aided UDA Positioning Accuracy

#### 2.3.1. Positioning Procedure

The procedures were performed by only one junior surgeon. In the test group only, Pirifix^TM^ was set, placed, and screwed around the piriform orifice before UDA separation. The screws used were 2 mm in diameter and 16 mm long for the upper fixings and 20 mm long for the lower fixings. Pirifix^TM^ was then removed while maintaining the same setting. 

Next, the connecting bridges were cut with pliers in each group. The mobile UDA and the mandible were attached to each skull using dental elastics (Southern Bald Eagle size ¼″ medium). The elastics were placed on hooks to reproduce muscular traction of the masseters, temporalis, medial pterygoid muscles, and palatine mucosa. The models were placed on the table using a fixing system.

Each UDA was then positioned according to the objectives set. Osteosynthesis was performed using modus 2 screws and titanium plates (Medartis, Basel, Switzerland). Positioning and osteosynthesis were performed twice per model. The first time, the UDA had to come back to its initial position. The second time, the UDA had to be positioned as the corrected and planned model. In each group, the vertical adjustment was set with a caliper in planned distances between the canine hooks and orbital rims.

In the control group, the first positioning was performed with a specific device. This one consisted of an initial position splint (P0 splint) and two spacers fitted into the osteotomy line. For the second positioning, we only used the occlusal splint (C splint) and the caliper. 

**Figure 7:**
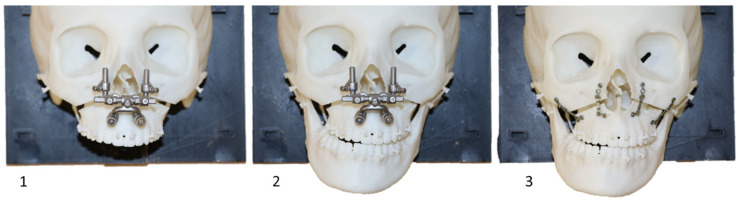
Operating procedure with Pirifix^TM^ (testing version) for movement C on model 14. 1: Repositioning of Pirifix^TM^; 2: positioning of the UDA; 3: osteosynthesis (the two lateral plates before the removal of Pirifix^TM^, the two medial plates after the removal of Pirifix^TM^).

In the test group, every positioning of the UDA was made only with Pirifix^TM^ using holes around the piriform orifice. The P0 positioning was dictated by using the device without modifying the pre-separation setting. The C positioning was implemented by unlocking the vertical axes to adjust the tilt and impaction of the occlusal plane (Figure 7). In the Pirifix^TM^ group, the osteosynthesis of the maxillo-zygomatic arches was performed first with Pirifix^TM^ in place. Pirifix^TM^ was then removed for paranasal osteosynthesis.

#### 2.3.2. Digitalizing

After UDA separation and simulated surgery, the printed models of each group were digitalized for the second and the third time to be compared to the corresponding planned models (P0 movement and C movement, respectively). The digitalizing and comparison protocol applied to each model was the same for both groups.

#### 2.3.3. Comparison of Precision

The accuracy of Pirifix^TM^ for placing the UDA in the planned and corrected positions was compared with the control group’s efficiency. The visualization tool with the color scale of CloudCompare software was used to identify the areas where the difference was the most important. The vertical dimension included upward/downward and tilting movements. The horizontal dimension included lateral translation and horizontal rotation. The sagittal dimension included forward movements and clockwise/counterclockwise rotations.

### 2.4. Statistical Analysis

No data were available in the literature to estimate the expected difference in accuracy between Pirifix^TM^ and occlusal splints. The number of subjects required could not be determined. Data were analyzed using R statistical v4.2.2 (The R Foundation for Statistical Computing, Vienna, Austria). The mean difference between the models of the two groups and the planned models was compared. Analysis was performed by applying a Student’s *t*-test or Wilcoxon Mann–Whitney test, depending on the number of models considered and variable distribution. Analyses were conducted as a two-sided test with 95% confidence intervals. The mean difference was considered significant if the *p*-value was less than 0.05. 

## 3. Results

### 3.1. Experimental Models

**Figure 8:**
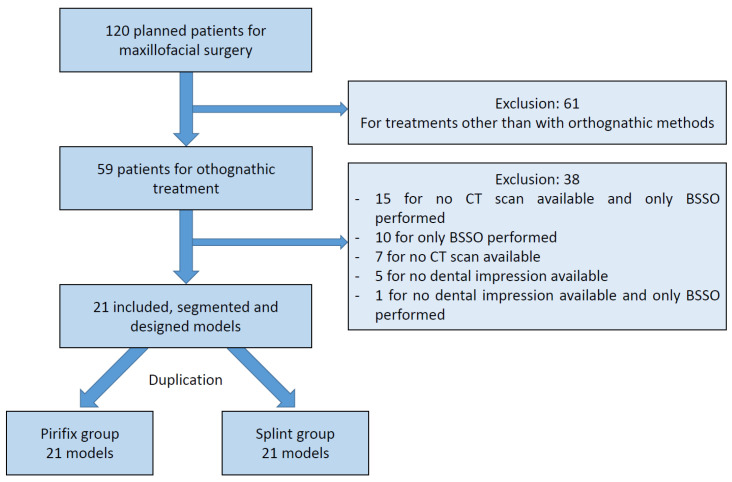
Flow chart. CT: computed tomography; BSSO: bilateral sagittal split osteotomy.

Data from 21 patients were included (Figure 8). The characteristics of the population are described in Table 1. Twenty-one skulls were segmented from DICOM and then designed. Two copies of each model were printed and divided into two groups.

### 3.2. Assessment of Group Comparability before the Separation of the UDA

Each model in the two groups was digitalized after printing and before UDA separation and compared to the pre-printed files. The mean difference between the pre-printed files and the digitalized printed models was 0.19 mm (+/− 0.10) in the control group and 0.21 mm (+/− 0.09) in the Pirifix^TM^ group (*p* = 0.313).

### 3.3. Evaluation of Device-Aided UDA Positioning Accuracy

**Figure 9:**
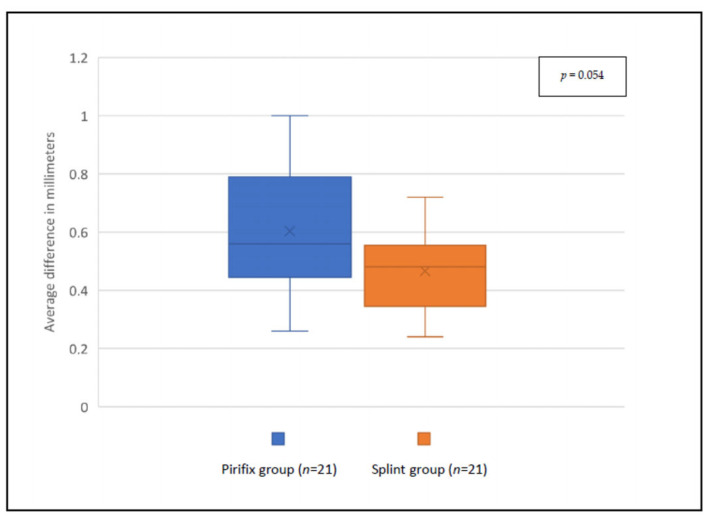
Comparison between planned files and printed models for P0 positioning in each group.

**Figure 10:**
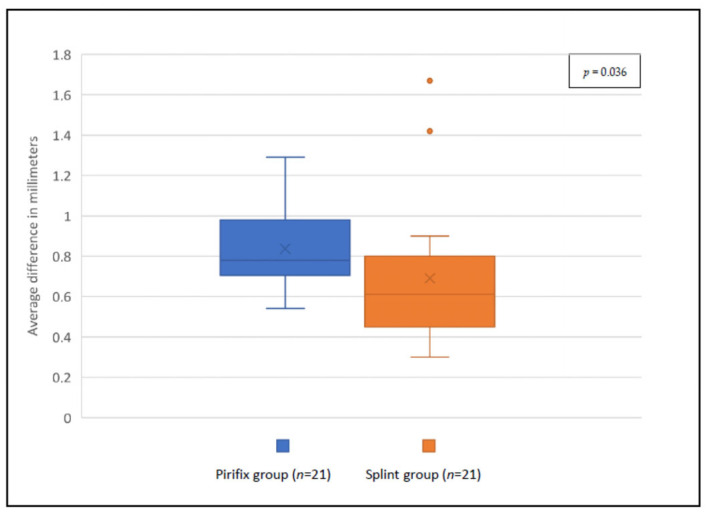
Comparison between planned files and printed models for C positioning in each group.

**Figure 11:**
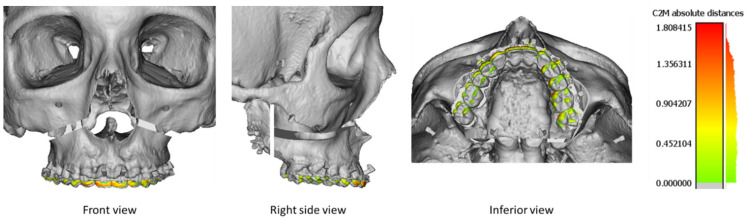
Superimposition between the 13 planned models (grey) and the 13 Pirifix^TM^-positioned upper dental arches (color) for the P0 position. The color scale shows the difference in mm and the distribution of the difference.

For the P0 position, the accuracy was 0.47 mm and 0.60 mm for the splint group and the Pirifix^TM^ group, respectively (*p* = 0.054) (Figure 9). For the corrected position, the accuracy was 0.69 mm and 0.84 mm for the splint group and the Pirifix^TM^ group, respectively (*p* = 0.036) (Figure 10 and Figure 11). In the Pirifix^TM^ for C positioning, 81.0% of models had an error in the vertical dimension, 61.9% in the sagittal dimension, and 66.7% in the horizontal dimension. In the splint group, this was 47.6%, 38.1%, and 76.2%, respectively. 

## 4. Discussion

### 4.1. Experimental Models

The model’s design meant that the osteotomy line had to be thick enough to perform rotational and translational movements without contact between the moving UDA and the upper osteotomy rim. The section was not intended to be a positioning guide.

The surface comparison of models was based on unmodified area matching. In their study describing a bone-and-teeth-supported surgical guide for LFIO, Kraeima et al. used zygomas, infraorbital margins, and foramen magnum [13]. Here, the UDA was subtracted from the model. The remaining file was also matched with the reference model. This provides a reliable assessment of the specific UDA-associated difference. Matching two whole objects would have superimposed them according to the smallest mean distance. In the event of an inhomogeneous difference, the difference would be smoothed over the entire model. This results in an increase in the observed difference of unmodified zones and an attenuation of the observed difference of zones that have been modified.

The comparability of both groups was ensured by the duplication of each model and their distribution in the two groups. Before the UDA’s separation, the average difference compared with the pre-printed file was not significant between the two groups. Several hypotheses may explain the difference in variability observed from one model to another. It may be due to a different placement on the printing plate (dictated by the size of the model) or by the presence of movements of the printed model on the rotating plate of the optical scanner during digitalization. 

### 4.2. Occlusal Splints

The 3D-printed occlusal splint was chosen as the reference method for two reasons. It remains a tried-and-tested method that has benefited from improved precision since 3D printing [10,23,24,25]. It is also less expensive and quicker to produce than custom-made titanium devices [14]. However, an occlusal splint is less accurate than surgical guides and custom-made osteosynthesis mesh [11,13,14]. 

### 4.3. Assessment of Pirifx^TM^

For the P0 position, precision was not significantly better in the splint group than in the Pirifix^TM^ group. The inaccuracy observed in the control group for the P0 position may be the result of incorrect condylar positioning or the persistence of residual print supports in the spacer zone. 

To prove the Pirifix^TM^ concept, we applied a movement combining horizontalizing and recentering the UDA. These movements were chosen for their larger aesthetic impact among both expert and non-expert observers [26]. For the C position, the accuracy of the UDA positioning performed by Pirifix^TM^ was significantly different from that of the splint group. The error observed in the splint group appears lower than in the Pirifix^TM^ group. 

These results suggest that the difference associated with the use of Pirifix^TM^ is related more to the settings of the axes than to its paranasal repositioning.

The difference visualization tool with color scale showed that the difference in the splint group was predominantly in the horizontal dimension. In the Pirifix^TM^ group, the error was more a combination of inaccuracy in the three planes of space, but the vertical dimension was predominant. This result seems consistent insofar as Pirifix^TM^ is a standard device that can be adjusted in all planes. For the C position, Pirifix^TM^ essentially performed horizontalization. It may explain that the difference is more important in the vertical dimension. The splint, on the other hand, allows for positioning in the horizontal plane and frontal or sagittal tilting in the occlusal plane, without being able to set vertical translation. In this group, the difference was more important in the horizontal dimension. The condylar position can be responsible for diduction with rotation in the transverse plane. Insufficient posterior blocking may also be responsible for errors such as clockwise rotation. The use of the visualization tool described the difference in each dimension without specifying the amplitude of the error. On this point, the two groups are also not comparable. 

In the Pirifix^TM^ group, positioning was assisted by the device but remained based on clinical criteria (only two measurements, assessment of centering and horizontalization). In addition, the device was fitted with clamping screws on a round-cut shaft, which could adversely affect the quality of locking. These factors may have influenced the results obtained in this group. The use of additional measures could improve the precision of the device. Furthermore, the models reproduced only the patient’s bone, whereas facial symmetry is influenced by facial soft tissue in clinical practice. 

The adaptability of Pirifix^TM^ was judged on the 21 included patients who had benefited from preoperative planning in the context of DSDy involving the midface. It was considered to have been achieved insofar as the adjustment of the device allowed for paranasal positioning without any identified conflicts. No bone conflicts were identified with the use of the device. Despite the anatomical diversity of the models, the number of patients is not enough to extrapolate the results to all midface disharmonies.

Among the MDs used for LFIO, Pirifix^TM^ offers a precise positioning alternative with a holding function that simplifies osteosynthesis. The splint allows for positioning in space with an error linked to a lack of predictability and reproducibility of the mandibular position. It can be used to adjust a tilt with an asymmetric thickness, but it cannot be used to adjust an upward or downward movement. These movements are associated with counterclockwise and clockwise rotation, respectively, due to mandibular rotation. The Pirifix^TM^ device is not custom-made and therefore has lower theoretical accuracy. However, it is supported on fixed bones above the osteotomy. This makes it more predictable, with a final inaccuracy of around 0.84 mm. The question arises as to the clinical relevance of precision in this context. Correct positioning must also be judged in terms of aesthetic consistency, involving facial soft tissues, and integration into the orthodontist’s treatment plan. 

The control group models were printed and operated on first. The results may be influenced by the learning process of the operator. This would apply in the handling of the caliper and the intraoperative taking of clinical measurements. All the models were operated on by a single surgeon. Feedback from many operators is needed to define the limits and improve the ergonomics of the device. A measurement bias is possible because the operator necessarily knows which device they have used for positioning. This bias was reproduced during the digitization and comparison phase since the same investigator was involved.

This is a preliminary study carried out on a 3D-printed simulator with no soft tissue. The aim was to prove the concept of this new medical device. Further investigations are still required, including facial soft tissue and technical feedback from other surgeons. The biocompatibility of the MD will have to be qualified before considering clinical trials. Pirifix^TM^ claims need to be assessed in studies conducted in clinical practice. The device is designed to simplify the positioning and maintaining of the UDA in the desired position during osteosynthesis. Its intraoperative adaptability makes it an attractive alternative to devices such as occlusal splints or custom plates. It remains to be seen how satisfactory the postoperative functional and aesthetic results will be. The invasiveness of the device, particularly when screwed around the piriform orifice, has yet to be assessed. The design of the lower parts seeks to avoid damaging the dental apexes. However, the design of the upper supports exposes patients to the risk of injury to the nasolacrimal ducts. Finally, the indications for its use need to be defined for a range of more or less complex disharmonies. 

## 5. Conclusions

Pirifix^TM^ is a new medical device. It is positioned as an alternative to other medical devices for positioning the upper dental arch during LFIO. It also claims to have a holding function to facilitate fixation. The Pirifix^TM^ concept seems to be producing encouraging results. There are many ways of optimizing the process by modifying design or production technology. The architecture of the device could make it possible to dissociate each translation and rotation movement. All parts could be miniaturized to reduce the overall dimensions. The use of Pirifix^TM^ for complex movements combining more than one rotation and one translation has yet to be investigated. Further studies are needed to determine how the device works with other bone anatomies, as well as with soft tissues. 

## Figures and Tables

**Table 1:** Population characteristics. Yo: years old; LFIO: Le Fort I osteotomy; BSSO: bilateral sagittal split osteotomy.

Characteristics		Data
Sex		
	Female	15 (71.4%)
	Male	6 (28.6%)
Age		
	<20 yo	5 (23.8%)
	20 to 30 yo	8 (38.1%)
	30 to 40 yo	3 (14.3%)
	>40 yo	5 (23.8%)
	Average	28.6 yo (16 to 55)
Angle’s classification		
	II	9 (42.9%)
	III	12 (57.1%)
Surgery		
	LFIO only	2 (9.5%)
	LFIO and BSSO	19 (90.5%)

## Data Availability

Data are contained within the article.
